# Master Regulators of Signaling Pathways: An Application to the Analysis of Gene Regulation in Breast Cancer

**DOI:** 10.3389/fgene.2019.01180

**Published:** 2019-12-03

**Authors:** Diana Tapia-Carrillo, Hugo Tovar, Tadeo Enrique Velazquez-Caldelas, Enrique Hernandez-Lemus

**Affiliations:** ^1^Computational Genomics Department, National Institute of Genomic Medicine (INMEGEN), Mexico City, Mexico; ^2^Graduate Program in Biological Sciences, National Autonomous University of Mexico (UNAM), Mexico City, Mexico; ^3^Center for Complexity Sciences, National Autonomous University of Mexico (UNAM), Mexico City, Mexico

**Keywords:** breast cancer, master regulator, signaling pathways, transcription factors, development

## Abstract

Analysis of gene regulatory networks allows the identification of master transcriptional factors that control specific groups of genes. In this work, we inferred a gene regulatory network from a large dataset of breast cancer samples to identify the master transcriptional factors that control the genes within signal transduction pathways. The focus in a particular subset of relevant genes constitutes an extension of the original Master Regulator Inference Algorithm (MARINa) analysis. This modified version of MARINa utilizes a restricted molecular signature containing genes from the 25 human pathways in KEGG's signal transduction category. Our breast cancer RNAseq expression dataset consists of 881 samples comprising tumors and normal mammary gland tissue. The top 10 master transcriptional factors found to regulate signal transduction pathways in breast cancer we identified are: TSHZ2, HOXA2, MEIS2, HOXA3, HAND2, HOXA5, TBX18, PEG3, GLI2, and CLOCK. The functional enrichment of the regulons of these master transcriptional factors showed an important proportion of processes related to morphogenesis. Our results suggest that, as part of the aberrant regulation of signaling pathways in breast cancer, pathways similar to the regulation of cell differentiation, cardiovascular system development, and vasculature development may be dysregulated and co-opted in favor of tumor development through the action of these transcription factors.

## Introduction

Breast cancers originate from healthy cells that are somehow reprogrammed to acquire unlimited proliferation and self-renewal capacity, among other properties, altogether referred to as "hallmarks of cancer" (Hanahan and Weinberg, 2011). These processes are the result of highly specific molecular interactions. In this context, it seems reasonable that cancerous cells make use of existing pathways through aberrant modulation mechanisms. Transcriptional regulation may play an important role in such altered mechanisms (Kolch et al., 2015).

Signal transduction pathways (STPs) are intricate molecular mechanisms that allow cells to sense specific signals, producing cellular actions in response and serve an important role in the integration of information (Dhanasekaran, 1998). Among these actions, the activation of transcription factors through STPs can modify the expression of genes with varying degrees of phenotypical effect.

STPs themselves can be regulated through the action of transcription factors (TFs) that modulate the transcription of groups of genes participating in them (Carroll and Brown, 2006; Laurent et al., 2015; Morgan and Pandha, 2017). As STPs are susceptible to external pharmacological modulation, an understanding of the regulation of the pathways may be helpful in the search for therapeutic targets (Steven Martin., 2003).

The analysis of the structure of a gene regulatory network that contains TFs and their targets, together with information of differential expression values, allows the identification of TFs with the greatest influence over the differences in expression. Those TFs are denominated transcriptional master regulators (TMRs). The Master Regulator Inference Algorithm (MARINa) (Lefebvre et al., 2010) can infer the TFs with greater influence in the transition between two conditions, as is the case with normal breast and breast cancer phenotype (Lefebvre et al., 2010; Tovar et al., 2015). In this work, we used a modified version of this algorithm to find the most important transcription factors associated with genes of known and well-curated signal transduction pathways obtained from the Kyoto Encyclopedia of Genes and Genomes (KEGG) (Kanehisa and Goto, 2000).

In breast cancer, where multiple transcription factors with hundreds or even thousands of targets are simultaneously deregulated, an integrative approach can help us to understand the biology underlying this disease. Modified MARINa analysis is complemented with the use of statistical enrichment analysis to look at the possible biological functions that are affected by the regulons of the TMRs (García-Campos et al., 2015). The information of biological knowledge databases was used (KEGG (Kanehisa and Goto, 2000) and Gene Ontology (GO) (Ashburner et al., 2000). Because the breadth of gene expression in the cell, the focus in a particular subset of processes or pathways has the advantage of giving us specific and less complex information about relevant processes and regulation in the cancerous phenotype.

## Materials and Methods

### Obtaining and Preprocessing Data

The Expression matrix was obtained from Espinal-Enriquez et al. (Espinal-Enríquez et al., 2017). The data corresponds to The Cancer Genome Atlas (TCGA) level 3 available data of the Illumina HiSeq RNA-Seq platform, and it consists of 881 samples (**Supplementary Datasheet 1**), of which 780 are from breast cancer tissue and 101 from adjacent healthy mammary tissue. Quality control and batch effect removal were performed with *NOISeq* (Tarazona et al., 2011) and *EDASeq* (Risso et al., 2011) R libraries, respectively (Espinal-Enríquez et al., 2017).

### Pathway Deregulation Analysis

To determine if signal transduction pathways are deregulated at the level of gene expression in our dataset of breast cancer, we estimated the degree of deregulation of KEGG Signal Transduction pathways by using the *Pathifier* algorithm (Drier et al., 2013). Pathifier assigns a score, denominated pathway deregulation score (PDS) for each pathway in a sample. For this, the expression status of the genes in the pathway is evaluated with reference to its expression in normal tissues of the same origin. In brief, for a given pathway, a multidimensional space is defined where each dimension represents the expression level of a gene. All samples are positioned in this space according to the expression levels of all the genes in the pathway. Then, a principal curve (a smoothed curve of minimal distance to all points) is calculated and all samples are projected into it. The score corresponds to the distance of the sample projection measured over the principal curve with respect to the projection of the normal tissue samples (Drier et al., 2013).

### The Master Regulator Inference Algorithm

**Figure 1:**
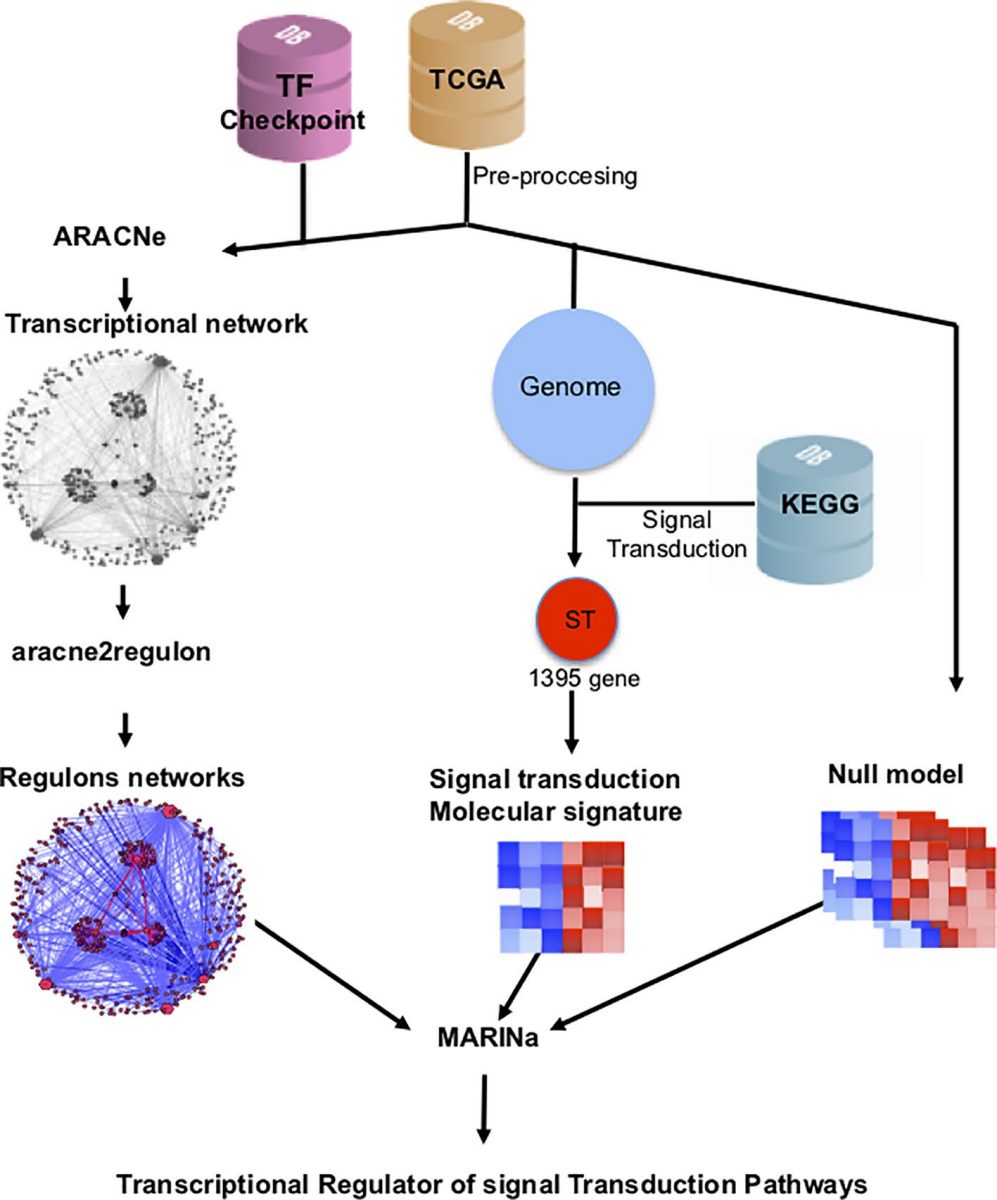
Customized MARINa pipeline. RNAseq data from TCGA's 780 invasive mammary carcinomas and 101 adjacent tissue samples was processed to obtain an expression matrix (*orange cylinder*). The expression matrix and a list of transcription factors from the TFCheckpoint database (*pink cylinder*) served as input to infer a transcriptional regulatory network with ARACNe. A regulon network was obtained associating the expression level of the targets of all transcription factors using the *aracne*2*regulon *function from *viper* (*left side*). For the generation of the molecular signature, we considered genes in the expression matrix in KEGG's "signal transduction" category (*blue cylinder*). Finally, a null model was generated by permuting sample labels and recalculating the molecular signature (*right*). These three elements are the input to MARINa for the inference of the transcriptional master regulators (TMRs) of the signal transduction pathways.

TMRs were inferred using the MARINa (Lefebvre et al., 2010). MARINa identifies TMRs through an enrichment of TF regulons (a TF with its targets) with differentially expressed genes between the two phenotypes (breast cancer vs. adjacent healthy mammary tissue). TMR inference with MARINa requires as input a network of regulons, a gene expression, molecular signature, and a null model (Lefebvre et al., 2010) (**Figure 1**). The construction of these elements is described below.

#### Generation of the Regulons Network

A regulon is defined here as a directed network where interactions describe regulatory interactions from a transcription factor to its transcriptional targets (TF Target). A regulons network is a regulon set, which is formed by the union of many regulons.

To obtain a regulon set from the data, we used the expression matrix of the tumor samples and a list of transcription factors in the TFCheckpoint curated database (Tripathi et al., 2013). From this database we selected those TFs that had experimental evidence for TF activity. A total of 771 of these TFs were expressed in breast cancer samples and present in the expression matrix (**Supplementary Datasheet 2**).

As a first step, transcription factors are associated with other genes expressed in the tissue. We used the mutual information-based algorithm ARACNe (Margolin et al., 2006) which calculates the pairwise mutual information for a pair of genes using the empirical probability distributions of their expression levels. For this network all possible interactions between TFs and genes in the expression matrix were calculated and kept if its *p* value was below 0.005.

Mutual information can detect both indirect and direct relationships. ARACNe constrains the number of indirect interactions applying the data processing inequality theorem (DPI), which considers that, in a triangle of interactions, the weakest one has a greater probability of being indirect if its difference is large with respect to the other two interactions (Hernández-Lemus and Siqueiros-García, 2013). We applied a DPI value of 0.2 as recommended in Margolin et al., 2006 (Margolin et al., 2006), which means that the weakest interactions of the triangles in the network were eliminated without introducing an excessive number of false positives.

The type of association (activation or repression) of the transcription factors is determined from the Spearman correlation of the TF with the levels of expression of all its targets (Lefebvre et al., 2010). This calculation was performed by the *aracne2regulon* function in the *viper* R package (Alvarez et al., 2016). This function transforms the undirected MI network from ARACNE into a regulons network that is directed.

#### Molecular Signature Generation of Signal Transduction Pathways

In the standard MARINa workflow, the molecular signature is built by comparing the expression level distributions of all genes between two conditions (e.g., healthy and diseased). For this work we built a molecular signature using only those genes annotated within the *signal transduction pathways* category in the KEGG database (Kanehisa and Goto, 2000). For the human species, this category contains 25 curated pathways. The total number of genes present in this subset is 1,700, of which 1,395 are included in the expression matrix (**Supplementary Datasheet 3**). The purpose of this filtering is to focus our search on those transcription factors that regulate the activity of these signal transduction pathways in breast cancer. The molecular signature was built by applying a *t*-test to each gene of the expression matrix, between tumors and adjacent healthy mammary tissue. The results of this test were *Z*-score normalized to allow comparability (Lefebvre et al., 2010).

#### Null Model Generation

To estimate the probability that a gene set enrichment score depends on the biological context and thus is not merely random, a null model was generated by random permutation between cases and control samples and recalculating differential expression values (Lefebvre et al., 2010).

### Inferring the Master Regulators of Signal Transduction Pathways

With the molecular signature, the regulon network and the null model, MARINa estimated the top regulons that enrich the most differentially expressed genes in the molecular signature through a gene set enrichment analysis (Subramanian et al., 2005). An additional constraint was to consider only TFs with 20 or more targets in the molecular signature (Lefebvre et al., 2010). A *p* value for each regulon was estimated by evaluating the enrichment score (ES) with reference to the distribution of scores of the null model (Lefebvre et al., 2010). For TMR inference we used *viper* package (Alvarez et al., 2016), an R implementation of MARINa available *via* Bioconductor.

The difference of this work with respect to MARINa lies in the use of a specific set of genes (signal transduction signature) which produces a ranking of the regulons for this specific subset. It is important to note that the regulons network used as input is the same as in regular MARINa, but the ranking is focused on the specific gene signature. The set of genes that constitute each regulon may include genes that are not in the molecular signature and can be part of a more diverse range of biological functions than signal transduction. This is the reason why we performed a subsequent enrichment analysis of the regulons with all KEGG human pathways.

### Functional Enrichment of the Top 10 Regulons Network With KEGG Pathways

An overrepresented pathway is defined as one for which we found significantly more genes within a test set than the number expected from a random sampling (García-Campos et al., 2015) hence, we say this set is enriched with genes of the pathway—this may in turn suggest biological relevance. The statistical significance of an enrichment can be assessed by means of an hypergeometric test. In order to know if the combined regulons of our top 10 transcription master regulators are enriched for biological pathways, an overrepresentation enrichment analysis (ORA) was performed using the WebGestalt web platform (Zhang et al., 2005) with KEGG as the functional reference database (Kanehisa and Goto, 2000). Statistical significance threshold was set to *p* = 0.05 after false discovery rate (FDR) correction.

The interrogation of the network for overrepresented pathways can evidence which of the original signal transduction pathways are being regulated. The genetic composition of the regulons is determined by the statistical dependencies between expression levels of the transcription factor genes and all other genes expressed in the tissue. Although we know the identity of the TFs, there is no guarantee that all transcription factor genes and the gene signature will be present in the network. This means that the clustering of the genes is not known *a priori* or imposed from a biological knowledge database like KEGG or GO. The co-expression of genes belonging to a function or pathway in different network modules has been previously observed (Alcalá-Corona et al., 2018).

### Regulon Enrichment of Gene Ontology Biological Processes

To gain insight on how our top 10 TMRs may contribute to this phenotype, we performed an ORA for each of the individual regulons with GO (Ashburner et al., 2000) biological process categories. GO was used because this database considers processes with various degrees of specificity, from very general processes expected in all cells to very specific subsets of a process (i.e., positive and negative regulation) and provides a reference that is independent from our original KEGG gene lists. GO biological process enrichments were calculated with WebGestalt (Zhang et al., 2005). Statistical significance threshold after FDR correction was set to *p* ≤ 0.05.

## Results and Discussion

### Pathway Deregulation Analysis

**Figure 2:**
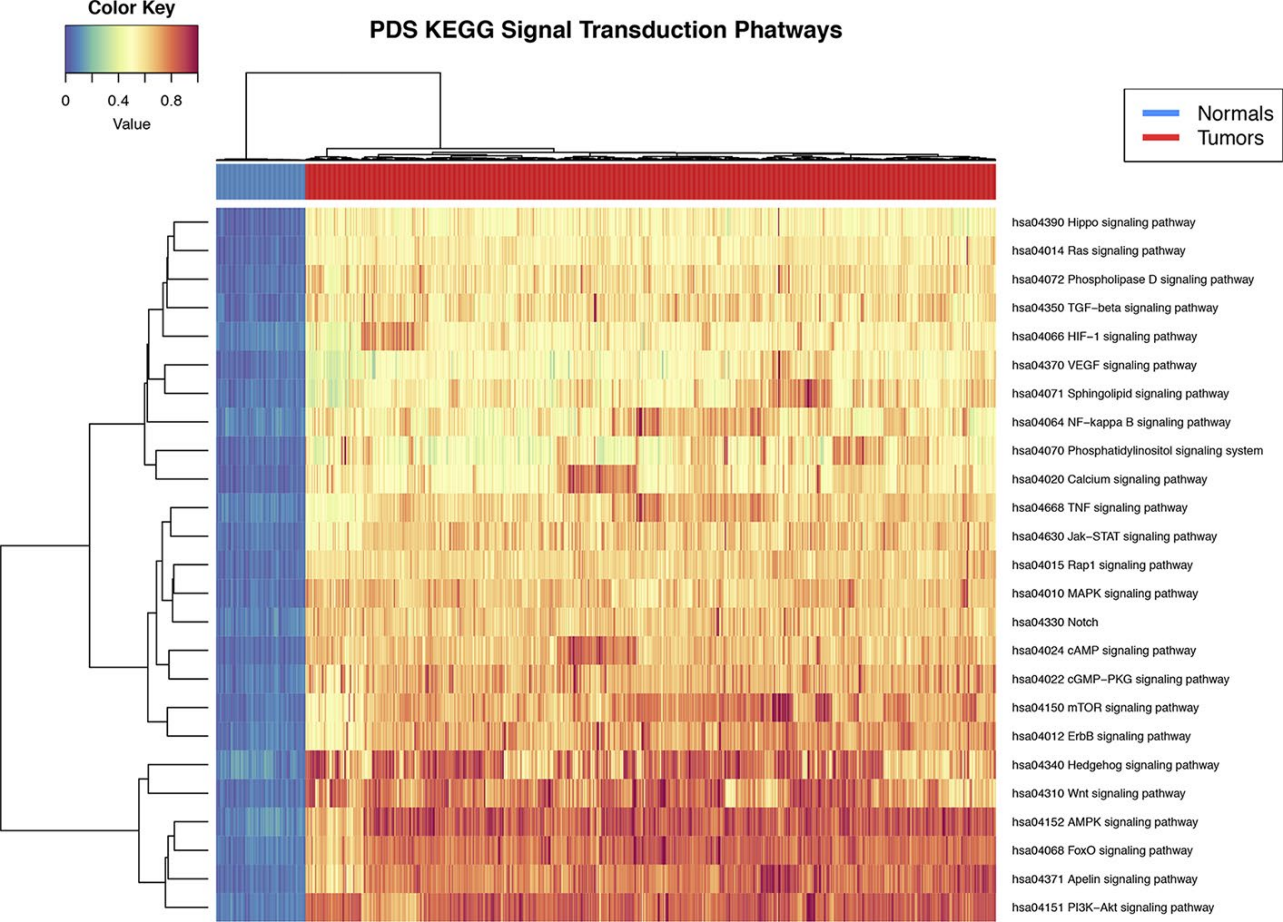
Heat map of pathway deregulation score (PDS) signal transduction pathways. In the *upper bar*, the control samples are shown in *blue* and the samples from breast cancer tissue in *red*. The color scale in the heat map represents the degree of deregulation by way of the healthy tissue samples. In the *upper left* is the color key in the values of PDS calculated from the sample by pathway.

Pathifier's pathway deregulation score is a measure of global difference in the expression levels of a set of genes compared to a reference. To determine if signal transduction pathways are deregulated at the level of gene expression in the breast cancer phenotype, we used the *Pathifier* algorithm (Drier et al., 2013). PDS are calculated for each sample and for each pathway. The deregulation analysis results show that all 25 signal STPs have a distinctive pattern in breast tumors with respect to normal breast tissue. This can be seen in the non-supervised clustering dendrogram at the top of **Figure 2** in the two major branches that early separate between both groups.

### Transcriptional Master Regulators of Signal Transduction Pathways

**Figure 3:**
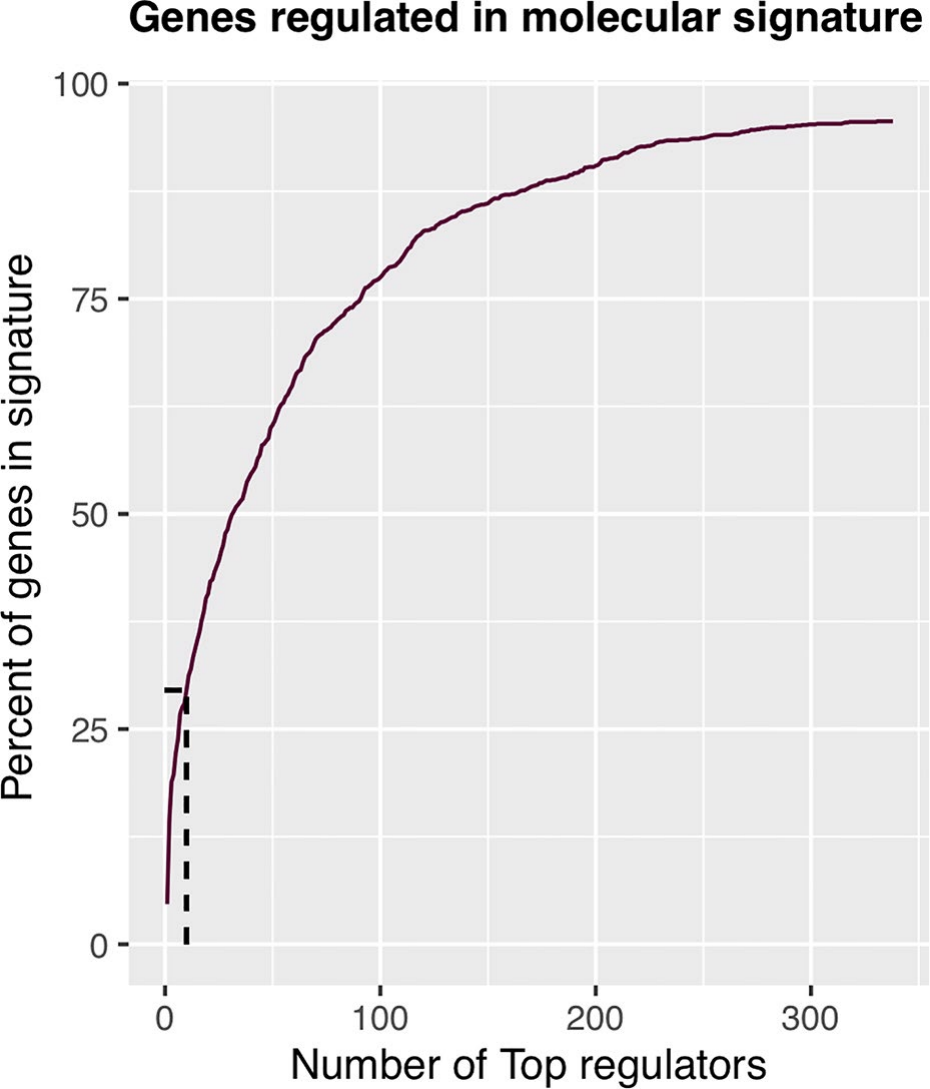
Percent of molecular signature genes regulated by master regulators. The first 10 master transcriptional regulators target about 30% of the genes belonging to the molecular signature. If we wanted to cover twice the genes belonging to the molecular signature, we would have to take five times more master transcriptional regulators; therefore, with 10 master regulators, the analysis is optimized.

**Figure 4:**
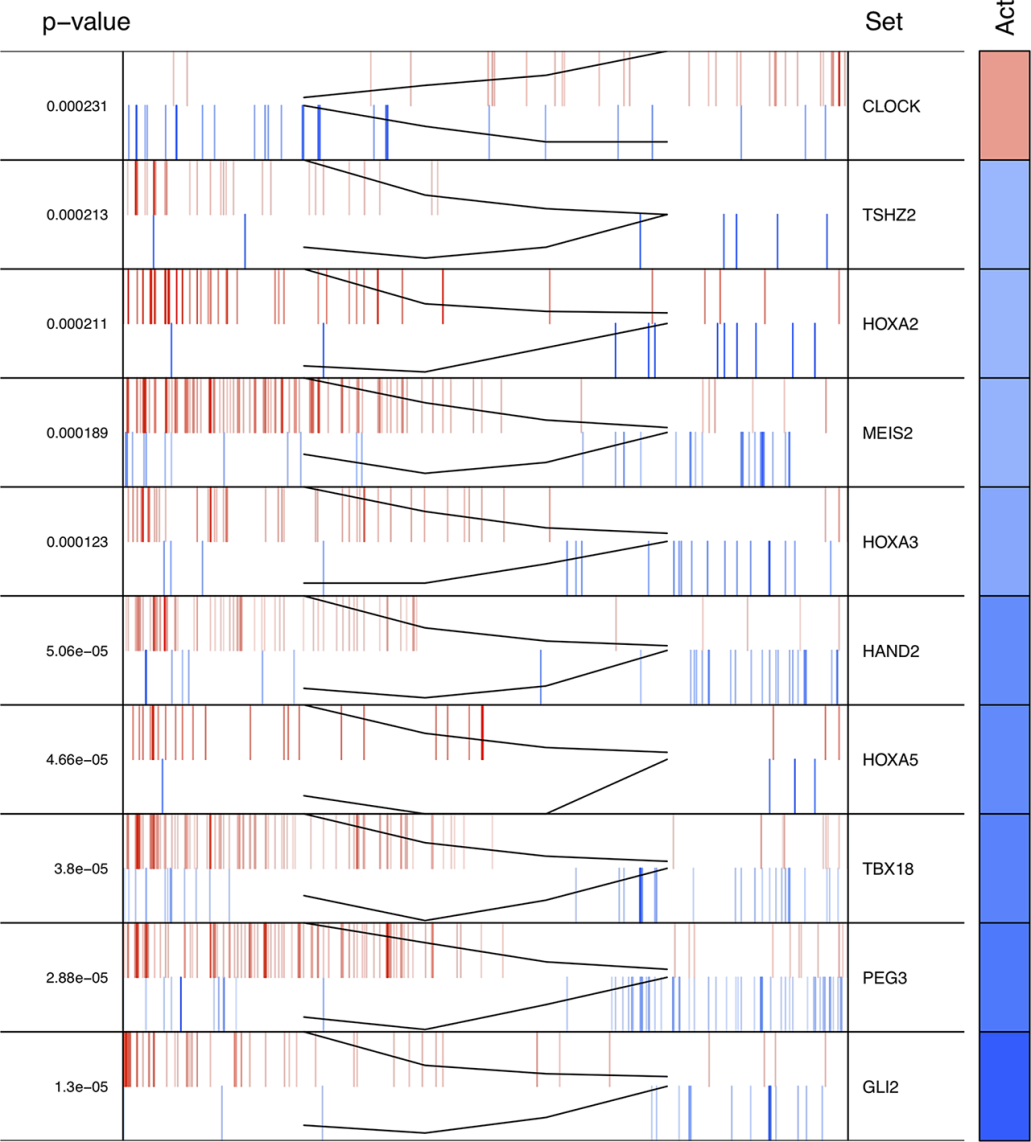
Top 10 master regulators of signal transduction pathways. These transcription factors control the genes of signal transduction pathways more differentially expressed in the tumor tissue. The total number of genes of signal transduction pathways (STPs) controlled by these regulons is 421, representing almost one third of the total genes in the molecular signature. The *first column* shows the enrichment *p* value for each regulon. The names of the master regulators are on the *right side*. The "Act" column indicates the activity of the master regulator on its STPs targets. *Red color* represents the over expression and the *blue color* represents the sub-expression with respect to normal tissue. *Barcode column* shows each gene in the molecular signature as one *vertical bar*. The *black line* on the *blue* and *red vertical lines* shows the walking down the ranked list of genes of the gene set enrichment analysis on the molecular signature genes.

The regulons network contains 765 TFs. From the TFs in the network, 338 met the requirement of having at least 20 targets in the molecular signature (**Supplementary Datasheet 4**). The output from MARINa contains a ranking of these regulons based on the integration of the regulons network structure and the differential expression of tumors with respect to normal tissue. In the ranking of the 338 TMRs, the top 10 TMRs regulate approximately 30% of the genes that belong to the set of the molecular signature (**Figure 3**). The top 10 master regulators of signaling pathways in breast cancer are: GLI Family Zinc Finger 2 (GLI2), Paternally Expressed 3 (PEG3), T Box 18 (TBX18), Homeobox A5 (HOXA5), Heart and Neural Crest Derivatives Expressed 2 (HAND2), Homeobox A3 (HOXA3), Meis Homeobox 2 (MEIS2), Homeobox A2 (HOXA2), Teashirt Zinc Finger Homeobox 2 (TSHZ2), and Clock Circadian Regulator (CLOCK) (**Figure 4**).

**Figure 5:**
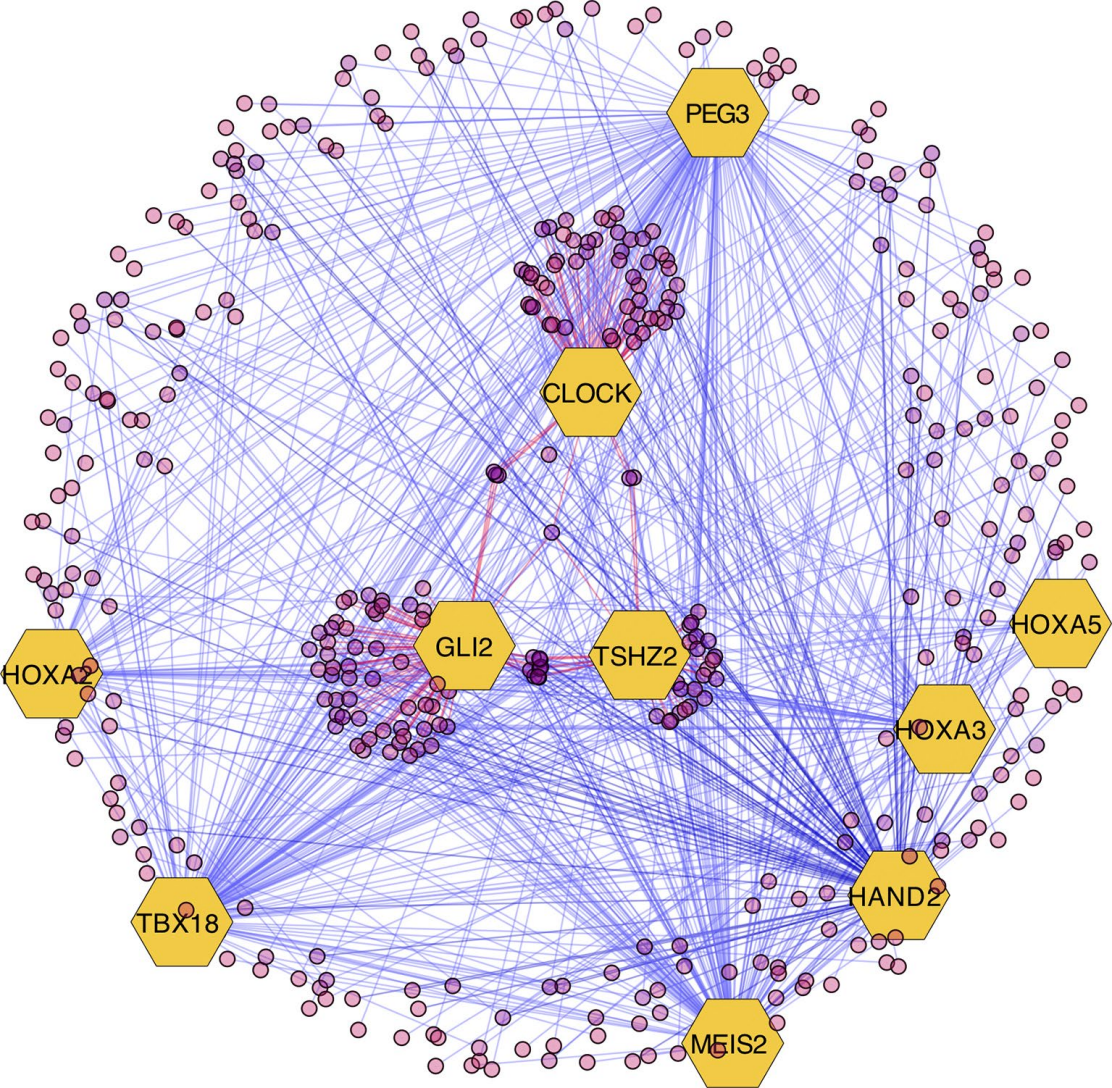
Visualization of the top 10 transcriptional master regulators (TMRs) (*big hexagons*) and their targets (*circles*). TMRs show a majority of repression interactions of their targets (shown as *blue links*) and comparatively few activation interactions (shown as *red links*). GLI2 is the TMR with the highest enrichment score (ES) of the top 10. Although GLI2 maintains activation interactions with some of its targets, the majority of its interactions are inhibitory. CLOCK is the only TMR that maintains a greater proportion of activation interactions [image generated with Cytoscape (Shannon et al., 2003)].

The results of the MARINa analysis show that, with the exception of CLOCK, the activity of these transcription factors over their targets is repression (Act column on the right side of **Figure 4**, negative values of NES in **Table 1**, and red links in **Figure 5**). Regulatory interactions in regulons are defined as activation if a target is overexpressed or inhibition if the target is unexpressed. The top 10 regulon network (**Supplementary Datasheet 5**) shows a higher proportion of repression interactions over their target genes (**Figure 5**). In this network, GLI2 is the only TMR interacting with more than one TMR (PEG3, TBX18, HAND2, HOXA3 HOXA2, and HOXA5). These genes, together with TSHZ2 and MEIS2, have been cited as transcription factors involved in morphogenetic processes like embryonic development and adult tissue remodeling like wound healing (Kuroiwa et al., 1996; Srivastava, 1999; Ruiz i Altaba et al., 2007; Melvin et al., 2013; Takeichi et al., 2013; Chojnowski et al., 2014; Amin et al., 2015; Machon et al., 2015; Jeannotte et al., 2016).

**Table 1:** Top 10 master regulators of signal transduction pathways.

Regulon	Size	NES	*p* value	FDR
CLOCK	59	3.68	2.31E−04	0.00592
TSHZ2	35	−3.7	2.13E−04	0.00592
HOXA2	54	−3.71	2.11E−04	0.00592
MEIS2	128	−3.73	1.89E−04	0.00592
HOXA3	67	−3.84	1.23E−04	0.00592
HAND2	93	−4.05	5.06E−05	0.00342
HOXA5	30	−4.07	4.66E−05	0.00342
TBX18	132	−4.12	3.80E−05	0.00342
PEG3	162	−4.18	2.88E−05	0.00342
GLI2	64	−4.36	1.30E05	0.00342

These transcription factors control the genes of signal transduction pathways more differentially expressed in the tumor tissue. With the exception of CLOCK, these regulators are commonly described within the context of embryonic development, and all of them have been reported in association with cancer. The total number of genes controlled by these regulons is 412, representing almost one third of the total genes in the molecular signature.

Most of the regulons of these TMRs are enriched with genes that are part of the Hedgehog Signaling pathway. Hedgehog is relevant during early morphogenesis and, in conjunction with Wnt, play a role in the self-renewal of stem cells (Reya et al., 2001). Both pathways have been previously described in cancer (Reya et al., 2001; Taipale and Beachy, 2001). Transcription factor TSHZ2 forms a complex with GLI1, which functions in a coordinated manner with GLI2 and GLI3 within the Hedgehog pathway (Riku et al., 2016). This was recovered from the regulons network in the form of gene expression associations. Additionally, a relationship with TBX18 and the Hedgehog pathway was previously reported in TBX18 knockout experiments where it showed a marked decrease in the expression of Hedgehog pathway genes (Wu et al., 2013).

GLI2 is the only TMR that shows multiple interactions with other TMRs (six in total; **Figure 5**). GLI2, together with GLI1, GLI3 (Ruiz i Altaba et al., 2007), and TSHZ2 (another of our TMRs) (Riku et al., 2016), are effector molecules activated within the Hedgehog pathway that modulate dedifferentiation and differentiation processes during early morphogenesis (Ruiz i Altaba et al., 2007; Scales and de Sauvage, 2009). This TMR is associated here in a context where the Hedgehog pathway is also represented.

### Regulons Network Enrichment of KEGG Pathways

The difference of this work with respect to MARINa lies in the use of a specific set of genes (signal transduction signature) which produces a ranking of the regulons for this specific subset of genes. The regulons network used as input is the same as in regular MARINa, but the ranking is constrained with the use of the specific gene signature. The sets of genes that constitute each regulon can vary in size from tens to several hundreds of genes and may include genes that are not in the molecular signature but are part of biological functions other than signal transduction. The statistical overrepresentation analysis allows the reduction of dimensionality from large lists of individual genes to fewer discernible biological functions, which is necessary given the large number of genes included in the network and the possibility of multiple annotations for each gene.

The top 10 regulons contain 4,052 genes associated by statistical dependencies. To know which pathways are most likely being regulated, we performed an overrepresentation analysis for all human KEGG pathways. This helped us to know which of the signal transduction pathways are represented, as well as other co-regulated pathways. The pathway with the most statistically significant enrichment was *Pathways in cancer* (hsa05200) with a coincidence of 121 genes. This pathway was not considered in the construction of the gene signature, although the enrichment is to be expected since it is a very large pathway composed of genes from many other signal transduction pathways and because we initially constrained our analysis to regulons that contained at least 20 genes of the molecular signature (see*Materials and Methods*).

Other pathways such as *Cell cycle* (hsa04110) and *Focal adhesion* (hsa04510) follow in the top 3 enrichments. Also enriched are signaling pathways present within our molecular signature and that are known to be important in the development of cancer, such as *PI3K-AKT signaling pathway *(hsa04151), *Phospholipase D signaling pathway* (hsa04072), and *Hedgehog signaling pathway* (hsa04340) (**Table 2**). As a whole, these pathways seem suggestive of coordinated signaling towards survival, proliferation, and differentiation, which are consistent with current knowledge of cancer biology. Some of the genes that are part of these regulons are known to take part in processes where cell growth and differentiation take place (i.e., morphogenetic processes). The functions and the possible relevance of these genes in the context of breast cancer are commented in the following sections.

**Table 2:** Enrichment analysis.

Enriched KEGG pathway	ID	FDR	No. of genes	Enrichment ratio
Pathways in cancer	hsa05200	0.000347	121	1.48
Cell cycle	hsa04110	0.00226	46	1.8
Focal adhesion	hsa04510	0.00386	66	1.58
Glioma	hsa05214	0.00993	27	1.98
Prostate cancer	hsa05215	0.0143	33	1.8
Huntington's disease	hsa05016	0.0148	60	1.51
PI3K-Akt signaling pathway	hsa04151	0.0148	96	1.37
Phospholipase D signaling pathway	hsa04072	0.0148	47	1.58
EGFR tyrosine kinase inhibitor resistance	hsa01521	0.0148	30	1.8
Hedgehog signaling pathway	hsa04340	0.0148	20	2.06

Statistical over representation analysis of KEGG pathways for the top 10 TMR regulons network was performed with Web Gestalt. The statistical significance threshold was set top ≤ 0.05 after FDR correction.

During the processes associated with tissue remodeling, signals such as morphogens, cytokines, and growth factors are present in the cell's environment. These activate signal transduction pathways that in turn induce changes within the cell (Christian, 2000). In the adult organism, tissue remodeling occurs after damage, or as part of very specific processes like lactation where the mammary gland structure changes dramatically (Hennighausen and Robinson, 2001; Macias and Hinck, 2012). This phenomena share a number of features, among which are cell proliferation, migration, and the formation of tissue structures like new blood and lymphatic vessels or epithelia. It is not unreasonable, though, that some components of the molecular machinery are similar between those processes and tumor development where similar structures are formed, although with abnormal results.

### Enrichment of Each Regulon in GO Processes

The most significantly enriched processes of each TMR regulon are presented in **Table 3**. In human breast cancer cells, HOXA5 was observed to activate the p53 tumor suppressor gene promoter (Raman et al., 2000). This agrees with the observation that the expression of HOXA5 in breast cancer cells expressing wild-type p53 led to apoptosis, while those lacking the p53 gene did not (Raman et al., 2000; Chen et al., 2004). Furthermore, the HOXA5 promoter region was methylated in 80% of p53-negative breast cancer specimens. (Raman et al., 2000). This aberrant regulation of HOX genes in breast cancer suggests that HOX genes may be important components in a network of gene regulatory mechanisms related to adult tissue homeostasis (Bhatlekar et al., 2014).

**Table 3:** Top significant enrichments of Gene Ontology biological processes per regulon.

Regulon	Enriched GO processes	ID	FDR	No. of genes	Enrichment ratio
CLOCK	Mitotic cell cycle	GO:0000278	1.39E−02	64	1.84
GLI2	Regulation of cell differentiation	GO:0045595	1.22E−05	73	2.07
HAND2	Cardiovascular system development	GO:0072358	4.31E−06	53	2.52
HAND2	Vasculature development	GO:0001944	4.31E−06	52	2.51
HOXA3	Tube development	GO:0035295	8.94E−05	46	2.5
HOXA5	Proximal/distal pattern formation	GO:0009954	1.69E−02	6	1.98
HOXA5	Anterior/posterior pattern specification	GO:0009952	1.69E−02	12	4.98
HOXA5	Skeletal system development	GO:0001501	1.69E−02	19	3.28
MEIS2	Animal organ morphogenesis	GO:0009887	5.29E−08	107	1.97
PEG3	Cell cycle	GO:0007049	8.72E−08	225	1.53
TBX18	Tissue development	GO:0009888	3.59E−05	139	1.61
TBX18	Blood vessel development	GO:0001568	3.59E−05	62	2.16
TSHZ2	Regulation of cell proliferation	GO:0042127	4.91E−02	45	1.92
TSHZ2	Extracellular matrix organization	GO:0030198	4.91E−02	17	3.32
TSHZ2	Extracellular structure organization	GO:0043062	4.91E−02	17	3.31

The top 10 regulons enrich more biological processes related to embryonic development. The statistical significance threshold was set top ≤ 0.05 after FDR correction.

Nine out of our 10 TMRs are recognized for taking part in morphogenetic processes (Kuroiwa et al., 1996; Srivastava, 1999; Ruiz i Altaba et al., 2007; Melvin et al., 2013; Takeichi et al., 2013; Chojnowski et al., 2014; Amin et al., 2015; Machon et al., 2015; Jeannotte et al., 2016). Enrichments of individual TMR regulons in GO biological processes recovered associations between TMR regulons with general morphogenetic processes.

Enriched GO biological processes are obtained from the molecular signature of the signal transduction pathways. The top places are occupied by processes associated with morphogenesis. These results are in line with the idea that tumors bear aberrations of growth, differentiation, and organization of cell populations. These are basic processes that are tightly coordinated and controlled during embryogenesis as well as in adult tissues. A similar idea has been previously proposed by Vinnitsky (1993), with the name of "oncogerminative theory of cancer development" (Vinnitsky, 1993). It suggests that cancer arises due to aberrant expression of developmental genes, and where the tumwor formation is a dynamic self-organizing process that effectively produces new tissue even if in an abnormal form. The malignant transformation of somatic cells, which can start with gene mutations combined with epigenetic dysregulation, can ultimately produce somatic cells reprogrammed into immortal cells that mimic germline cells. These mimics are termed "cancer stem cells" or "oncogerminative cells" (Vinnitsky, 1993; Bhatlekar et al., 2014).

Enrichments of GO biological processes for each individual regulon in the top 10 show a recurrent theme in processes associated with morphogenesis (Kuroiwa et al., 1996; Srivastava, 1999; Ruiz i Altaba et al., 2007; Melvin et al., 2013; Takeichi et al., 2013; Chojnowski et al., 2014; Amin et al., 2015; Machon et al., 2015; Jeannotte et al., 2016). These results are in line with the idea that tumors bear aberrations of growth, differentiation, and organization of cell populations. Although our results cannot assure the activity of morphogenetic processes in tumors, there is an association at the level of gene expression patterns of molecules canonically represented in them.

### HOXA Family Enriched in Regulons

**Figure 6:**
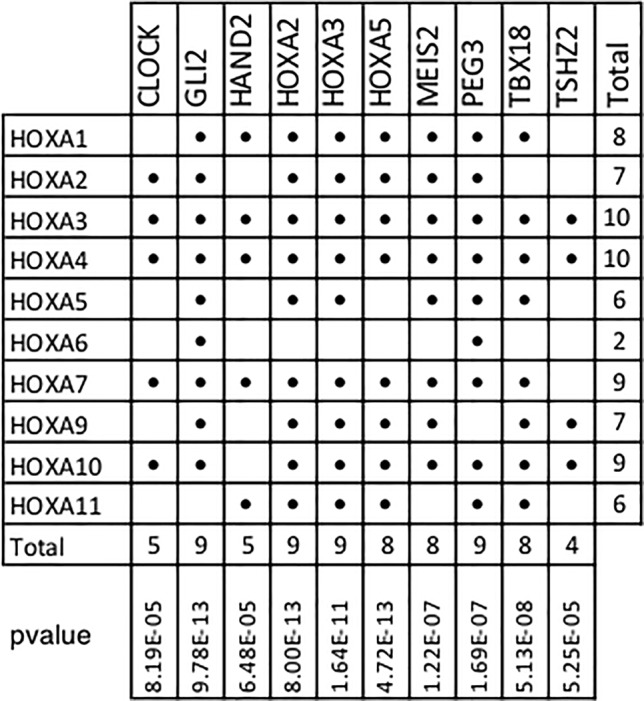
HOXA family genes present in top transcriptional master regulator (TMR) regulons. Numerous HOXA family members are part of the top TMR regulons and a significant *p* value was found in all cases. Hypergeometric test parameters are: population size, *N* = 15,802 genes in the expression matrix; number of successes in the population, *M* = 10 of 11 human HOXA genes in expression matrix; sample size, *s* is the regulon size and number of successes and *k* is the number of HOXA genes present in the regulon. It is noteworthy that the only human HOXA gene not found in the regulons of all the master transcriptional factors detected by MARINa (338) was HOXA13.

In addition, our results show that 10 of the 11 members of the Homeobox A family are included within the top 10 TMR regulons (**Figure 6**). In humans, HOXA consists of 11 genes (HOXA1, HOXA2, HOXA3, HOXA4, HOXA5, HOXA6, HOXA7, HOXA9, HOXA10, HOXA11, and HOXA13). Although the HOXA genes code for proteins with transcription factor activity, these are not typically considered as components of signal transduction pathways. HOXA TFs act not only as transcriptional activators in cancers but also as transcriptional repressors (Ladam and Sagerström, 2014); thus, both upregulation and downregulation of the members of this family may be relevant in carcinogenesis. Many HOXA genes (HOXA1, A2, A3, A5, and A9) have been shown to have significantly lower expression levels in cancerous tissues compared to non-cancerous ones.

## Conclusions

With the generation of gene regulatory networks and a molecular signature centered on signal transduction pathways, we present a list of genes and their transcriptional regulators that may be modulated signal transduction pathways in breast cancer. This information may be helpful in the study of this disease where pathway analysis showed that all pathways from KEGG in this category are deregulated in a large dataset of breast cancer samples.

We identified TSHZ2, HOXA2, MEIS2, HOXA3, HAND2, HOXA5, TBX18, PEG3, GLI2, and CLOCK as the top 10 TMRs regulating signal transduction pathways in breast cancer. These genes regulate 30% of the genes in these pathways. CLOCK is the only TMR in the top 10 that shows a positive regulation of its predicted targets, while the other top TMRs show negative regulation relationships, although the molecular mechanisms by which those TMRs act should be explored in future studies.

The enrichment analysis of the top 10 TMR regulons recovers information about processes that are well recognized in cancer (angiogenesis, organogenesis, proliferation, survival, etc.). These results are reassuring in the sense that we know we are recovering relevant biological information from the phenotypes under study instead of random associations. Furthermore, the analysis of individual regulons allows the identification of specific molecules that may be playing key roles. This seems to be the case with genes in the HOXA family, which are within the top 10 TMRs and as part of regulons. Genes of this family are known for their role in morphogenetic processes as well as in the maintenance of adult tissues and with altered expression in tumors.

We present a modified version of MARINa that utilizes a specific gene signature with genes in KEGG's Signal Transduction category. The reason for this is to narrow the search of TMRs to those most relevant to signal transduction pathways. This approach, however, is not limited to a particular pathway or process and the molecular signature can be modified to reflect other research questions relative to specific pathways or processes.

Signal transduction pathways serve an important role as information integrators in the cell. Components and their interactions are thus of great interest and subject of a thorough study. Furthermore, signal transduction pathways are susceptible to external pharmacological modulation. An understanding of the regulation of the pathways may be helpful in the search for therapeutic targets.

## Data Availability Statement

Publicly available datasets were analyzed in this study. Data comes from TCGA/GDC public repositories. The list of GDC samples used in this work is in the **Supplementary Datasheet 1**. The list of transcription factors with experimental evidence obtained in TFCheckpoint.org is in the **Supplementary Datasheet 2**.

## Author Contributions

DT-C implemented methods, performed calculations, analyzed results, and drafted the paper. HT implemented methods and analyzed and discussed the results. TV-C contributed to the discussion and drafting of the paper. EH-L designed and supervised the study, contributed to the analysis of the results and discussion, and revised the manuscript. All authors read and approved the final version of the manuscript.

## Funding

This work was supported by CONACYT (grant no. 285544/2016), as well as by federal funding from the National Institute of Genomic Medicine (Mexico). Additional support has been granted by the Laboratorio Nacional de Ciencias de la Complejidad (grant no. 232647/2014 CONACYT). EH-L is the recipient of the 2016 Marcos Moshinsky Fellowship in the Physical Sciences.

## Conflict of Interest

The authors declare that the research was conducted in the absence of any commercial or financial relationships that could be construed as a potential conflict of interest.
